# A Study of Machine-Learning Classifiers for Hypertension Based on Radial Pulse Wave

**DOI:** 10.1155/2018/2964816

**Published:** 2018-11-11

**Authors:** Zhi-yu Luo, Ji Cui, Xiao-juan Hu, Li-ping Tu, Hai-dan Liu, Wen Jiao, Ling-zhi Zeng, Cong-cong Jing, Li-jie Qiao, Xu-xiang Ma, Yu Wang, Jue Wang, Ching-Hsuan Pai, Zhen Qi, Zhi-feng Zhang, Jia-tuo Xu

**Affiliations:** ^1^Department of Basic Medical College, Shanghai University of Traditional Chinese Medicine, 1200 Cailun Road, Pudong New Area, Shanghai 201203, China; ^2^Shanghai Collaborative Innovation Center of Health Service in Traditional Chinese Medicine, Shanghai University of Traditional Chinese Medicine, 1200 Cailun Road, Shanghai 201203, China

## Abstract

**Objective:**

In this study, machine learning was utilized to classify and predict pulse wave of hypertensive group and healthy group and assess the risk of hypertension by observing the dynamic change of the pulse wave and provide an objective reference for clinical application of pulse diagnosis in traditional Chinese medicine (TCM).

**Method:**

The basic information from 450 hypertensive cases and 479 healthy cases was collected by self-developed H20 questionnaires and pulse wave information was acquired by self-developed pulse diagnostic instrument (PDA-1). H20 questionnaires and pulse wave information were used as input variables to obtain different machine learning classification models of hypertension. This method was aimed at analyzing the influence of pulse wave on the accuracy and stability of machine learning model, as well as the feature contribution of hypertension model after removing noise by K-means.

**Result:**

Compared with the classification results before removing noise, the accuracy and the area under the curve (AUC) had been improved. The accuracy rates of AdaBoost, Gradient Boosting, and Random Forest (RF) were 86.41%, 86.41%, and 85.33%, respectively. AUC were 0.86, 0.86, and 0.85, respectively. The maximum accuracy of SVM increased from 79.57% to 83.15%, and the AUC stability increased from 0.79 to 0.83. In addition, the features of importance on traditional statistics and machine learning were consistent. After removing noise, the features with large changes were h1/t1, w1/t, t, w2, h2, t1, and t5 in AdaBoost and Gradient Boosting (top10). The common variables for machine learning and traditional statistics were h1/t1, h5, t, Ad, BMI, and t2.

**Conclusion:**

Pulse wave-based diagnostic method of hypertension has significant value in reference. In view of the feasibility of digital-pulse-wave diagnosis and dynamically evaluating hypertension, it provides the research direction and foundation for Chinese medicine in the dynamic evaluation of modern disease diagnosis and curative effect.

## 1. Introduction

Hypertension is a clinical syndrome whose principal characteristic is an increase in systemic arterial pressure and it is the one of the most common cardiovascular diseases in the world [1–3]. According to 2017 Chinese guidelines for the management of hypertension [4], the prevalence of hypertension has been increasing in China for decades, reaching 23.2%, which has greatly affected the health status of people. In addition, hypertension and other cardiovascular diseases, whose prevention cannot be ignored, possess the characteristics of high incidence, high mortality, and heavy medical burden.

 At present, the data on the classification and prediction of hypertension mainly come from inpatient electronic medical records, environmental and genetic factors, and gene expression data [5]. Zhiyong Pei [6] used support vector machine (SVM) to classify and predict by inputting environmental factors, genetic factors variables, and environmental and genetic factors for 559 hypertensive patients and 641 healthy people. It was found that the combination of environmental variables and genetic factors would improve the prediction accuracy, reaching 80.1%. Sherif Sakr [7] used cardiorespiratory fitness data and combined the different machine learning methods with the SMOTE algorithm to achieve the higher prediction accuracy of hypertension at 81.69% with AUC of 0.93. In short, there are some achievements on studies in the classification and prediction of hypertension, but these researches have some limitations to some extent. On one hand, they focus on the prediction of a certain stage of data and the preconditions are relatively numerous. On the other hand, the subjectivity of the data is relatively strong. Most of all, data features rarely involve the pulse wave-based hypertension prediction studies.

As one of the methods of traditional Chinese medicine (TCM) diagnosis, pulse diagnosis has always played an important role in clinical diagnosis and treatment and subhealth recuperation [8]. Since the 1950s, the integration of biomedicine, mathematics, physics, biomechanics, bioengineering, computer science, and traditional Chinese medicine has made great progress in the objective study of pulse diagnosis. The pulse wave reflects the shape of the pulse beats and pulse graph is used as the objective image for recording the pulse wave. By combining with the medical history, lab-test diagnosis, and four-combined Chinese medicine diagnosis, and analyzing the correlation of pulse graph with the disease and syndrome, the study gradually establishes the clinical standard diagnosis of pulse graph, which further provides the objective diagnostic method for clinical practice in Chinese medicine. Pearson's correlation coefficient and t-test are often applied for analysis of time domain or frequency domain features, either of which is the classical analysis method in pulse wave [9]. In addition, Hu [10] used KNN to undertake the classification prediction of the pulse wave of the elderly people with different blood pressure segments, but in this article she did not conduct the modelling prediction for multiple age groups. The age of the samples is mostly elderly, and the model has certain limitations. Zhang [11] used genetic algorithm to screen features, and then SVM predicted the time domain information of pulse wave, which obtained accurate rate and ROC of 76.0% and 0.83, respectively. Many studies have suggested that pulse waves have strong correlation with hypertension [12–15].

The research of pulse wave digital processing also has obtained better results and has been applied to the objectification of pulse diagnosis in TCM [16–18]. Based on this, some machine learning methods such as support vector machine, neural network, and random forest [19] have been used in pulse wave recognition or classification. Although some achievements have been made in pulse wave recognition and classification, there are still some problems, such as poor prediction accuracy and model stability, and fuzzy characteristic contribution in the hypertension model. The reason for this may be some deficiencies in model building and the impact of data noise.

Clustering analysis [20] is a classical method of unsupervised learning, and its most representative algorithm is K-means. We attempt to classify different samples into different groups based on K-means clustering analysis method and sort out the contribution of clustering according to the sample characteristics, and then make further analysis after removing the low-quality samples. Additionally, cardiovascular monitoring commonly relies on sphygmomanometers and less on the risk prediction of chronic cardiovascular disease. The assessment of cardiovascular dynamic risk is more comprehensive by combining pulse wave and H20 scale [8, 19, 21] with symptom assessment.

The main purposes of this paper are as follows: (1) to remove the noise of pulse wave by K-means, so as to further improve the accuracy and stability of the model; (2) to identify the feature variable which is of the highest contribution to hypertension.

## 2. Materials and Methods

### 2.1. Subjects

A total of 929 subjects were collected from the outpatient department and the medical examination center of Shanghai Shuguang Hospital attached to Shanghai Chinese Medicine University affiliated to Shanghai University of Traditional Chinese Medicine. Among them, 450 (356 males and 94 females, average age: 44.73 ± 8.73) were diagnosed with hypertension and 479 (337 males and 142 females, average age: 44.49 ± 9.18) with no hypertension.

### 2.2. Inclusion and Exclusion Criteria

Inclusion criteria for hypertensive group were as follows: patients who met the diagnosis standards of hypertension [4] with the age ranging from 18 to 70, male or female.

Inclusion criteria for healthy group were as follows: (1) the examination results of B-ultrasound, electrocardiogram, biochemistry, imaging, and other subjects in physical examination indicate no disease; (2) the age ranged from 20 to 70, and gender is not limited; (3) H20 score is greater than 80 with no positive items; (4) systolic blood pressure of 90-140mmHg and diastolic pressure 60-90mmHg.

Exclusion criteria include (1) those diagnosed with other serious medical conditions, such as cardiovascular and cerebrovascular diseases, urinary tract diseases, tumors, and immune and hematological diseases; (2) those who suffered from mental illness; (3) those who refused to sign the informed consent; (4) those whose later screening data are not qualified.

### 2.3. Pulse Image Collection and Analysis Methods

#### 2.3.1. Collection Instrument

**Figure 1:**
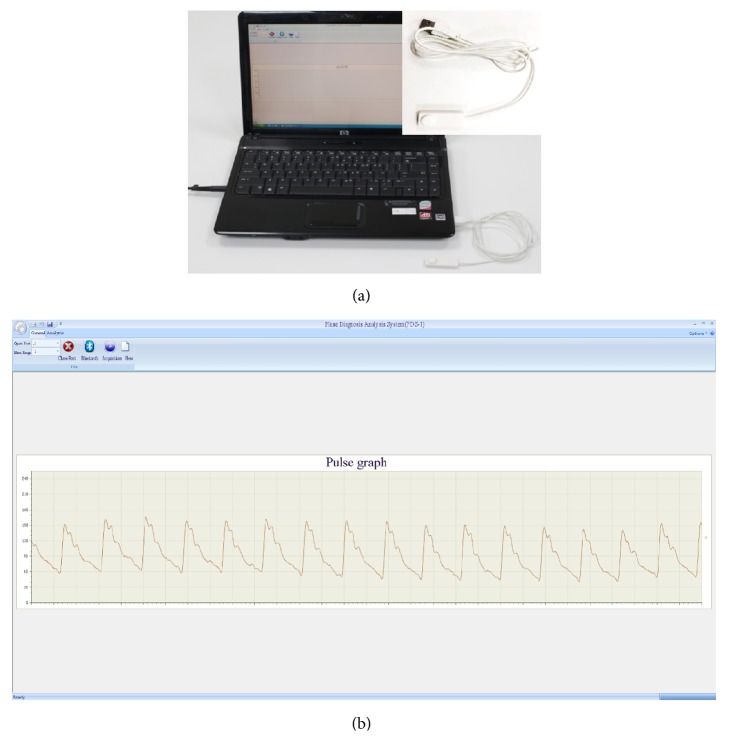
Collection instrument. (a) Pulse diagnosis instrument (PDA-1). (b) Interface of pulse diagnosis and analysis system.

In this study, a pulse diagnosis instrument (PDA-1) (Figure 1(a)) was developed by “Traditional Chinese Medicine Diagnosis Information Intelligent Processing Research Team” in Shanghai University of Traditional Chinese Medicine (SHUTCM). The device consists of a pulse wave transducer, an A/D analog-to-digital transducer, and a computer. The main technical parameters are sensitivity (0.5mV/gram force; linear range: 0-250 grams force; output impedance: 1 K special symbol), AC amplifier loop (input dynamic range: 0-25mV; full-scale output ±5V), and DC amplifier loop (input dynamic range: 0-125mV; full-scale output ±5V).

#### 2.3.2. Collection Methods

The pulse diagnosis instrument (PDA-1) was used to collect the pulse wave of radial artery on the left hand and the patients were asked to sit still or rest for at least 5 minutes before the acquisition. During the collecting process, patients were required to sit or stay supine, relax, not talk, and breathe normally. If the above requirements were not satisfied, second collection was needed.

**Figure 2:**
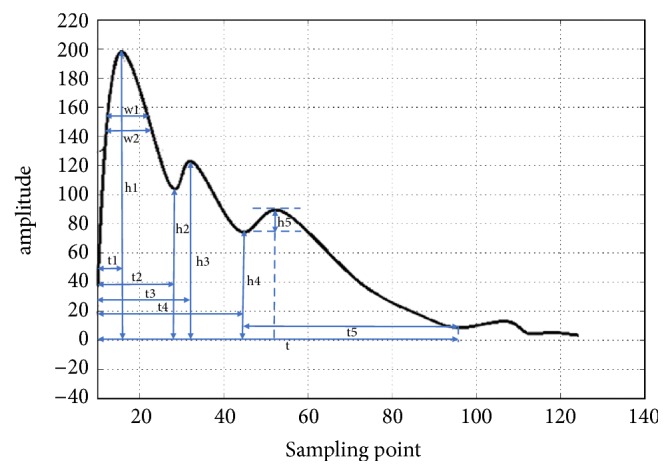
The main measurement parameters of pulse wave cycle.

In order to obtain the pulse wave parameters, the Intelligent Information Processing Laboratory of Chinese Medicine Diagnostics of Shanghai University of TCM has developed the method into a pulse diagnosis and analysis system (PDAS) (Figure 1(b)). The system menu has “General" and “Analysis." “General" includes “Open Port," “New," and “Acquisition." “Analysis" includes “Generate Report," “Calculate Characteristic Value," “Export H Value," and “Batch Analysis." The data view window shows pulse wave information. The section at the lower right shows the pulse wave of the patient with hypertension (Figure 1(b)). The software outputs the information parameters of pulse for subsequent analysis. Time domain features of the pulse wave [10, 22, 23] including 6 duration features (t, t1, t2, t3, t4, and t5), 5 amplitude features (h1, h2, h3, h4, h5, h1/t1, h3/h1, and h4/h1), 4 width features (w1, w2, w1/t, and w2/t), and 2 area features (As, Ad) were extracted by Shannon energy envelope and Hilbert transform [24]. The meaning of the features is listed in Table 1 and Figure 2.

### 2.4. Study Design and Setting

**Figure 3:**
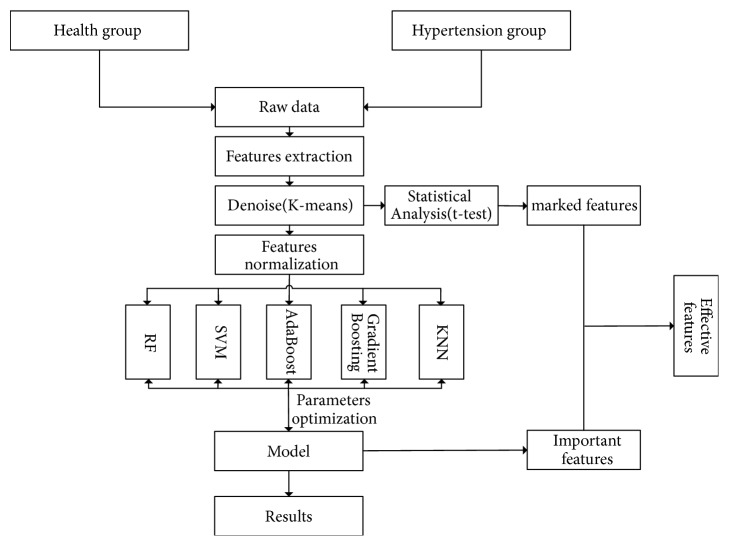
Flow chart.

Features such as age, BMI, pulse wave parameters, and H20 score of the hypertension and healthy groups were inputted as independent variables in the pretreatment. Subjects were categorized according to whether they have hypertension (dependent variable). 60% of the samples were for training examples and 40% were for testing examples. See flow chart (Figure 3).

#### 2.4.1. Noise Reduction

The K-means algorithm is a partition-based clustering algorithm. A dataset is divided into several groups or classes. Data with higher similarity is in the same group while dissimilar data is in different group. Cluster analysis can help to find abnormal data because similarity and dissimilarity are based on the attribute of data. Similar or neighboring data are aggregated to form each cluster set, and those data, outside these cluster sets, are to be excluded. During the process of data collection, human factors interfered with the data inevitably. Clustering analysis is used to cluster the input feature variables to obtain different levels of clustering results. In a word, cluster analysis is clustered based on different feature variables, and similar samples are gathered into one group.

Firstly the K-means algorithm determines the reference value k and then divides the N data again in the k clusters, so that clusters with a similar degree in each cluster are classified into one and clusters with a low degree of similarity are classified into another. Specific steps: First, any number of k data in the dataset were found. The original centroid of each cluster was represented by these data. Second, the remaining datasets were divided into each cluster according to the minimization principle, which was based on the distance between each dataset and its cluster centroid. Finally, the centroid of each cluster was calculated again. The above operation was repeated and the calculation was stopped when the value of the objective function was minimum [25].

#### 2.4.2. Feature Normalization

Owing to the differences in the magnitude of the parameters, it has a negative effect on the classification and prediction. The range is scaled and mapped from 0 to 1 (or -1 to 1 if there are negative values).

The formula of MinMaxScaler is

(1) $$\begin{array}{c} \frac{x_{i}-\min\left(x\right)}{\max\left(x\right)-\min\left(x\right)} \end{array}$$

where min⁡(x) is the minimum value, max⁡(x) is the maximum value, and *x*_*i*_ is the value for each feature.

#### 2.4.3. Classifier

Random Forest (RF), Support Vector Machine (SVM), AdaBoost, Gradient Boosting (GBT), and K-Neighbor (KNN) are classical machine learning models. SVM, AdaBoost, and RF are widely used as classification predictions. Support vector machine is a sparse kernel machine, which is a model that only relies on data subset (support vector) to predict unknown class labels [26, 27]. Based on the support vector machine theory, it is pointed out that, for a nonlinear separable dataset containing two classes of points, there are many hyper planes used to classify the classes. And a common radial basis function was chosen. SVM is used to classify training samples to separate and optimize two classes of hyper planes (i.e., decision boundaries). The optimal decision boundary between support vectors is chosen by the distance of the maximum boundary M [28]. Support vector machines have good generalization capabilities. In other words, the decision surface is seen as linear in the high-dimensional space, while it is considered as nonlinear in low-dimensional feature space, which means that SVM could be applied to nonlinear separation data. In addition, in terms of overfitting problems, support vector machine is of robustness for high-dimensional data [29]. The main drawback is that it is more difficult to interpret the generated model and has a certain sensitivity to appropriate parameter adjustments.

K-nearest neighbor (KNN) is one of the easiest methods to predict classification in pattern recognition [29]. To obtain the nearest neighbor for each dataset, KNN uses measurement to calculate the distance between data pairs. In general, the measurement used is Euclidean distance. Since each new data point is classified differently, KNN can establish a local approximation of the objective function [30]. While a test example is classified, it will use a similarity function based on the Euclidean distance to find training examples of the K most recent query points [29]. Since high k results in overfitting and model instability, the appropriate values must be specifically chosen [28]. Another advantage of KNN is its simplicity. In spite of this, the forecasting time is usually very expensive because all the training data must be reexamined [30].

Random Forest (RF) uses a majority vote to predict categories based on data partitions from multiple decision trees [28]. In each decision tree, data points fall into specific leaves according to their characteristics and are assigned a forecast. Then the data points are averaged. The maximum number of voting categories will provide the final forecast [26]. The Gini index is used to determine the “best split" threshold for a given category of input values. Compared with the parent node, the Gini index returns the measure of the heterogeneity of the child nodes [28].

AdaBoost is a supervised learning algorithm for solving classification problems [31]. In each sequence, misclassified instances are given more weight for the next sequence while correctly classified instances are given lower weight. The final model is a linear combination of all the models created in the previous sequence [32]. In addition, GBDT has very few limitations and assumptions on the input data, so it is very flexible to deal with complex nonlinear relationships [33]. In some problems, it is more stable than other learning algorithms and is less susceptible to be affected by overfitting problems. Each learning algorithm has its advantages and tends to be more suitable for certain types of problems than other types of problems, and there are usually many different parameters and configurations that need to be adjusted before achieving the best performance of the dataset [34]. Gradient Boosting could strategically combine some simple tree models to obtain optimized predictive performance while the result model could be interpreted by identifying key variables [33]. The core is that the learning objective of each subtree is the residual of the previous subtree. The sum of all subtrees can be used as the final result of the model. GBT can handle different types of predictors and missing data. At the same time, it does not need to eliminate outliers and perform previous data transformations [35].

SVM is used mainly to establish a classification hyper plane as the decision surface and maximize the isolated edges of the positive and negative instances and then construct and solve the optimization problem by selecting appropriate kernel functions and appropriate penalty parameters. AdaBoost is an iterative algorithm, whose core objective is to train different classifiers for the same training set (i.e., weak classifiers) and then combine weak classifiers into a stronger classifier. Besides, K-Neighbors uses the distance calculation method. According to the new data calculated by all features and categorical distance of data point in the dataset, it will operate classification prediction by sorting them in ascending order of distance.

#### 2.4.4. Parameters Optimization and Evaluation Criteria

Due to the different performances of different models, different classification models of hypertension were constructed, respectively. At the same time, different machine learning was performed by grid search and 10-fold cross-validation to optimize the parameters. The optimal parameters were selected to establish the model.

In order to assess the feasibility of the above methods, analysis was performed using common evaluation criteria [36], including accuracy rate (ACC), area under the ROC curve (AUC), sensitivity (ST), and specificity (SP). AUC [37], an evaluation binary model, is one of the popular methods. AUC is used in the range from 0 to 1. Moreover, the four basic statistical definitions which describe the process of classification are TP (true positive, number of positives), FP (false positive, number of negatives), TN (true negative, number of negatives), and FN (false negative, number of positives).

(2) ACC=TP+TNTP+FP+FN+TN×100%

(3) Sensitivity=TPTP+FN×100%

(4) Specificity=TNFP+TN×100%

### 2.5. Development Platform

Statistical analysis was performed using SPSS 22.0 software. The BMI, age, and pulse wave parameters were analyzed in two groups by an independent samples t-test. The data were shown as mean and standard deviation. P <0.05 indicates a statistical difference.

The data was collected and analyzed by python3.5 and sklearn [38] to achieve machine learning. Orange3.11 [39] was used for cluster analysis and removing unqualified pulse wave.

## 3. Result

### 3.1. Noise Reduction

**Figure 4:**
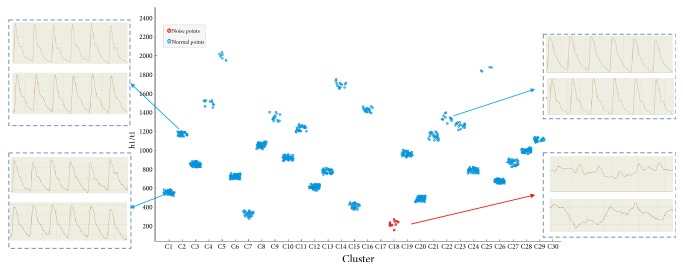
k-means clustering analysis. The x-axis represents the number of clusters while the y-axis represents the variable h1/t1. Red points include noise pulse wave. Blue points include normal pulse wave.

Studies [40, 41] have shown that h1/t1 can reflect the ability of cardiac ejection and aortic compliance; then elasticity and compliance of vascular directly affect blood pressure. According to the results of the k-means in Figure 4, the points in the red circle below could be considered as the group with the most noise, which indicates that the pulse wave of hypertensive population was better for noise recognition. After clustering, it was found that one group contains more noise and the other group contains less noise. Pulse waves whose noise was removed are classified into one group and those whose noise was not removed are in the other group to conduct the comparative analysis. h1/t1 had greater difference in different pulse waves. The pulse wave with greater similarity was gathered into one group, whereas the pulse wave with the large noise was collected into another group in the red circle shown in Figure 4.

### 3.2. Between Hypertension and Healthy Group of the Pulse Graph Characteristics

The results of pulse wave parameter between the healthy group and the hypertension group after noise reduction are shown in Table 2. Compared with the healthy group, the hypertension group has higher BMI, h1, h2, h3, t2, t5, h1/t1, w1/t, and HR and lower h5, t, w2/t, H20 score, and Ad (P<0.05).

### 3.3. Five Data Mining Algorithms Classification

The study used RF, SVM, AdaBoost, Gradient Boosting, and K-Neighbors to establish a hypertension identification model based on pulse wave features and results were shown in Table 3. Through the comparison of different machine learning algorithms, it could be seen that the accuracy of the four kinds of machine learning models had been improved in comparison to the result of the pulse wave analysis after noise removal, and SVM had the largest increase. Although the accuracy rate of K-Neighbors had also greatly improved, the accuracy rate of prediction was the lowest. At the same time, in terms of classifier performance, the biggest increase in AUC was SVM and AdaBoost. By observing the accuracy and stability, AdaBoost, Gradient Boosting, and RF had better result, and K-Neighbors classifier had unsatisfactory result.

**Figure 5:**
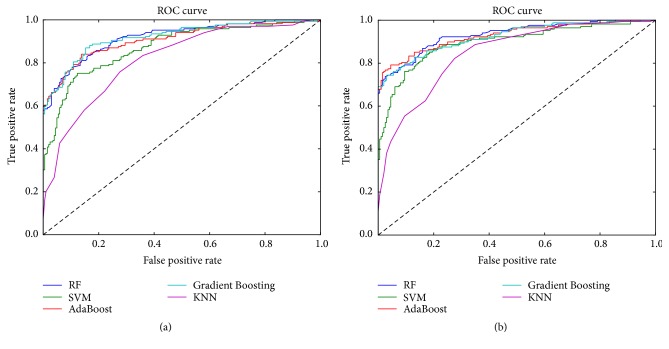
The ROC curve in different model. (a) Data without noise reduction. (b) Data with noise reduction. The x-axis denotes false positive rate. The y-axis is true positive rate. In Figure 5, the dark blue line represents Random Forest (RF). The green line represents support vector machine (SVM). The red line represents Adaboost. The light blue line represents Gradient Boosting. The purple line represents K-nearest neighbor (KNN).

The ROC curve is a graph that describes the performance of the binary classifier system. In other words, the ROC curve is plotted based on true positive rate and false positive rate. Sensitivity is also known as TPR, which means that the possibility of high blood pressure is truly judged. Specificity is equal to the true negative rate, which means that there is no possibility of disease. The area under the ROC curve is most commonly used as an accurate index. If the sensitivity and specificity reach 1, the area under the ROC curve reaches the desired accuracy. The best prediction method generates a point in the upper left corner (0, 1) of the ROC space, representing 100% sensitivity (no false negatives) and 100% specificity (no false positives). In this study, sensitivity, specificity, and ROC (AUC) are used to evaluate the classifier performance. As shown in Figure 5, the ROC curve in the figure is a classifier result using a noise reduction and no noise reduction dataset, respectively. Different colored lines represented the ROC curve of different machine learning models. Among them, AdaBoost and Gradient Boosting had the most significant changes in the noise reduction and non-noise reduction curves, which indicates that the AdaBoost and Gradient Boosting classifiers have higher sensitivity and specificity after noise reduction.

### 3.4. Feature Importance

**Figure 6:**
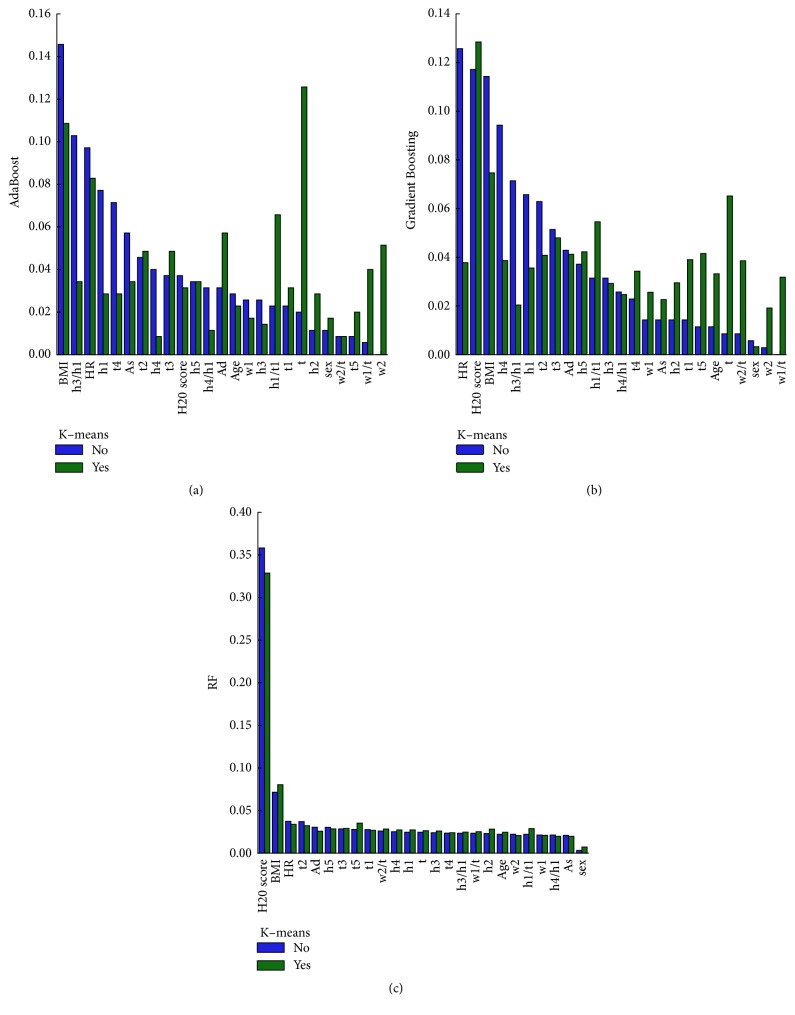
The importance of the classification variables features. The bar charts (a), (b), and (c) represent the results of feature importance of AdaBoost, Gradient Boosting, and Random Forest (RF), respectively. The y-axis represents the value of the importance for variables features. Note that “yes" represents that K-means is used to reduce noise in this model and vice versa.

The variables output by the machine learning model were compared and analyzed to obtain the degree of contribution of the model. The results on the three models are shown in Figure 6.

Compared with the results of classification after reducing noise, the features of the hypertensive classification model varied greatly among different machine learning. AdaBoost and Gradient Boosting had the most significant changes, and RF had the smallest. After removing the noise in the AdaBoost and Gradient Boosting models, the importance of h1/t1, w1/t, t, w2, h2, t1, and t5 variables (top10) had increased. Among them, AdaBoost had the most prominent changes in w2, w1/t, t, h1/t1, h2, t5, and Ad. In RF, only BMI, t5, h2, and h1/t1 variables had increased.

**Figure 7:**
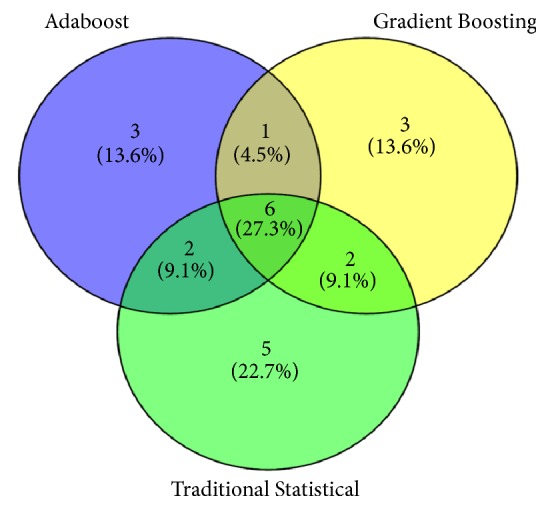
The analysis of the importance on the features of traditional statistics and machine learning.

Furthermore, compared with the features of significant difference in traditional statistics, machine learning AdaBoost and Gradient Boosting had significant difference in the common feature rankings. Therefore, AdaBoost and Gradient Boosting were selected to analyze the important features. The variables of the top 12 are listed in Table 4. In Figure 7, the common variables among the three are h5, t, Ad, BMI, h1/t1, and t2, which indicates that hypertension may play an important role in cardiac output.

## 4. Discussion

Previous studies showed [12–15] that hypertension and pulse waves had a strong correlation, and statistical description of this study also confirmed this phenomenon. The comparative results of the pulse wave characteristics showed that the values such as h1/t1, h1, h3, and w1/t were higher than those in the healthy group. Therefore, the differences of characteristics in pulse wave between hypertensive and healthy group made it possible to further classify them using machine learning.

However, in terms of the analysis of raw data, filtering pulse wave lacks the methods of quality control. At present, there are human interference factors in the acquisition process of pulse wave, so it is necessary to reduce the noise. Taking advantage of pulse waves after noise reduction as the input variables of the machine learning, the study suggests that the machine learning accuracy and stability have significantly improved.

Figure 4 shows clustering analysis of data. Different sample clustering had found a certain degree of regularity. Pulse waves with higher heat values were grouped together (typical hypertension pulse wave); pulse waves within middle-ranged heat values were collected together (mixed part of hypertension pulse wave and healthy pulse wave); pulse waves with low heat values were gathered together. Further observation revealed that the pulse wave with a lower heat value was mixed with more noise. This part of the noise was in line with the pulse wave neither in hypertensive group nor in healthy group. This noise primarily derives from respiration and muscle tension. On the one hand, the respiration can lead to abnormalities in the pulse wave. On the other hand, subjects who have the high muscle tension are likely to cause tremor of the pulse wave. Therefore, the elimination of such pulse wave is necessary.

Table 3 shows the classification results of pulse waves with noise reduction. The classifications were obtained by the RF, SVM, AdaBoost, Gradient Boosting, and K-Neighbors algorithms, respectively. Meanwhile, 10-fold cross-validation and grid optimization were performed to measure the classification performance. Evaluation indicators pointed out that, compared with the results of pulse wave classification without noise reduction, AdaBoost and Gradient Boosting had the better classification effect after noise reduction, and SVM had the larger increase.

Significant features obtained from traditional statistical analysis are specific, such as h1, h2, h3, t2, t5, h1/t1, w1/t, h5, t, w2/t, Ad, H20 score, and BMI, but traditional statistical analysis is weak in linking nonlinear relationships. Machine learning methods, however, have advantages in this regard. Through traditional analysis and machine learning analysis, the prediction results are relatively satisfactory, but machine learning cannot see the specific operation mechanism of “black box.” With the development of technology over the years, some machine learnings become valuable reference on practical use. The results showed that there were significant differences in the importance ranking on features among three different machine learnings before and after noise reduction. Among them, AdaBoost and Gradient Boosting had the better changes in feature importance. The importance of variables features in t, t1, h2, h1/t1, t5, w1/t, and w2 had ascended to some extent. Therefore, the result suggests that the wave of wiry pulse is more obvious after noise reduction and this is consistent with the theory of traditional Chinese medicine [10].

The application of the combination of TCM diagnostic technology and modern information technology is promising. The development of the wearable Chinese medicine pulse wristband represented by the pulse modernization research is based on the traditional Chinese medicine theories combined with modern information technology, artificial intelligence, and other technologies. In addition, it also retains the characteristics of pulse diagnosis in TCM, including miniaturization, wearable, wireless transmission, and intelligence, and it can be widely used in Chinese medicine teaching, scientific research, medical care, health care, and many other fields, with broad domestic and international market prospects. Currently, there is a wearable health assessment technology based on pulse evaluation, which provides a new method for further disease prevention and evaluation [40]. The effective development of wearable Chinese medicine pulse diagnosis bracelets and the transformation into products will greatly promote the development and application of modern Chinese medical diagnosis and treatment technologies and bring many positive social and economic benefits. On one hand, portable pulse acquisition provides objective results and data indicators for clinical diagnosis and therapeutic evaluation. On the other hand, it provides technical basis for the modern clinical research of TCM with Chinese characteristics and conforms to the connotation of TCM. Finally, pulse diagnosis is a TCM diagnostic technology information. This study lays a foundation for further exploration on wearable pulse diagnostic equipment.

## 5. Conclusion

Based on the pulse wave, this paper used cluster analysis to eliminate noise and machine learning to establish a classification model for hypertension. It shows good classification effect and indicates that removing noise has great significance in improving accuracy and stability of model. It also illustrates that it is feasible to use computer technology to conduct TCM diagnosis. Besides, it is also part of establishing the classification model to identify the factors that affect hypertension diagnosis. The results of traditional analysis and machine learning imply that the variables of h1/t1, h5, t, Ad, BMI, and t2 are likely to connect with hypertension.

Through collecting and analyzing the information of hypertension, this study explores the information and application of traditional Chinese medicine and provides a reference for the design of a more effective classification model of hypertension. In addition, combined with the symptoms and signs of the patients and the information of tongue and pulse diagnosis in Chinese medicine, the development of a more convenient and wearable pulse diagnostic instrument provides a more real-time, convenient, and quick method for further study in the prevention and prediction of hypertension.

## Figures and Tables

**Table 1:** TD features.

No.	Features	Meaning
1	h1	Main wave amplitude. It reflects the compliance of the aorta and the cardiac ejection function of the left ventricular

2	h2	Main isthmus wave amplitude. Same physiological significance as h3.

3	h3	Heavy wave front wave amplitude. It reflects the elasticity of arterial vessels and its peripheral resistance.

4	h4	Dicrotic notch amplitude. It reflects the peripheral resistance of arterial vessels and the closure of aortic valve.

5	h5	Gravity wave amplitude. It reflects the compliance of the aorta and the function of aortic valve.

6	t1	Left ventricular rapid ejection period. The time value from the start point to the crest point of the main wave on the pulse graph.

7	t2	The duration of the beginning of the tidal wave.

8	t3	The duration of the crest of the tidal wave.

9	t4	Left ventricular systolic duration. The time value from the start point to the dicrotic notch on the pulse graph.

10	t5	Left ventricular diastolic duration. The time value from the dicrotic notch to the end point on the pulse graph.

11	t	Includes left ventricular systolic and diastolic duration. The time value from the start point to the end point on the pulse graph.

12	w1	main wave 1/3 height. The duration of maintaining high intravascular pressure.

13	w2	main wave 1/5 height. The duration of maintaining high intravascular pressure.

14	w1/t	The ratio of the width of the main wave at its 1/3 height to the entire pulse cycle. It reflects the proportion of the duration time of continuous high pressure in the aorta in the entire pulse cycle.

15	w2/t	The proportion of the duration time of continuous high pressure in the aorta in the entire pulse cycle.

16	h1/t1	cardiovascular function

17	As	Systolic area. The area on the pulse graph is related to cardiac output.

18	Ad	Diastolic area.

**Table 2:** Comparison of the variables between hypertension group and healthy group ($\overset{-}{X}\pm S$).

**Feature**	**Healthy group**	**hypertension group**	**p value**
**Age**	44.44 ± 9.204	44.7 ± 8.706	0.37
**BMI**	23.91 ± 2.961	25.55 ± 3.306	0.0*∗∗*
**w1**	0.18 ± 0.035	0.18 ± 0.033	0.55
**w2**	0.13 ± 0.034	0.14 ± 0.034	0.05
**As**	0.22 ± 0.029	0.22 ± 0.028	1
**Ad**	0.11 ± 0.035	0.1 ± 0.036	0.0*∗∗*
**h1**	116.0 ± 35.992	126.11 ± 44.893	0.0*∗∗*
**h2**	84.98 ± 32.215	93.18 ± 40.319	0.02*∗*
**h3**	78.02 ± 29.356	85.73 ± 36.019	0.01*∗∗*
**h4**	44.78 ± 15.047	47.55 ± 18.016	0.09
**h5**	12.8 ± 4.455	12.16 ± 4.056	0.04*∗*
**t1**	0.14 ± 0.021	0.14 ± 0.022	0.31
**t2**	0.24 ± 0.037	0.24 ± 0.041	0.0*∗∗*
**t3**	0.27 ± 0.033	0.27 ± 0.038	0.08
**t4**	0.36 ± 0.03	0.36 ± 0.034	0.23
**t5**	0.41 ± 0.023	0.41 ± 0.028	0.0*∗∗*
**t**	0.85 ± 0.119	0.83 ± 0.128	0.01*∗∗*
**h1/t1**	838.82 ± 276.686	919.03 ± 355.812	0.0*∗∗*
**h3/h1**	0.67 ± 0.128	0.68 ± 0.133	0.36
**h4/h1**	0.39 ± 0.082	0.38 ± 0.082	0.11
**w1/t**	0.21 ± 0.036	0.22 ± 0.033	0.0*∗∗*
**w2/t**	0.16 ± 0.036	0.16 ± 0.035	0.0*∗∗*
**HR**	77.08 ± 9.613	80.4 ± 11.839	0.0*∗∗*
**H20 score**	85.74 ± 4.868	76.38 ± 10.331	0.0*∗∗*

Compared with healthy group. *∗* P <0.05, *∗∗* P <0.01.

**Table 3:** Results on the classification of machine learning model.

Model	ACC	AUC	SP	ST
RF	0.841	0.832	0.936	0.728
RF*∗*	0.853	0.848	0.905	0.792
Gradient Boosting	0.852	0.843	0.941	0.746
Gradient Boosting*∗*	0.864	0.859	0.920	0.798
SVM	0.796	0.792	0.833	0.752
SVM*∗*	0.832	0.828	0.865	0.792
AdaBoost	0.839	0.830	0.921	0.740
AdaBoost*∗*	0.864	0.858	0.925	0.792
KNeighbors	0.729	0.716	0.852	0.580
KNeighbors*∗*	0.736	0.728	0.830	0.625

Models with an asterisk *∗* mean that K-means is applied before using these models.

**Table 4:** Selected (top 12) features in model.

model	Feature variable	Feature importance
Adaboost	t	0.126
	BMI	0.109
	HR	0.083
	h1/t1	0.066
	Ad	0.057
	w2	0.051
	t2	0.049
	t3	0.049
	w1/t	0.040
	h5	0.034
	h3/h1	0.034
	As	0.034

Gradient Boosting	H20 score	0.128
	BMI	0.075
	t	0.065
	h1/t1	0.055
	t3	0.048
	h5	0.042
	t5	0.042
	Ad	0.041
	t2	0.041
	t1	0.039
	h4	0.039
	w2/t	0.039

## Data Availability

The datasets generated and analyzed during the current study are not publicly available due to the confidentiality of the data, which is an important component of the National Key Technology R&D Program of the 13th five-year plan (No. 2017YFC1703301) in China, but are available from the corresponding author on reasonable request.
